# DRP1, fission and apoptosis

**DOI:** 10.1038/s41420-025-02458-0

**Published:** 2025-04-07

**Authors:** Nan Wang, Xinwai Wang, Beiwu Lan, Yufei Gao, Yuanyuan Cai

**Affiliations:** 1 The Department of Neurosurgery, China-Japan Union Hospital of Jilin University, Changchun, China; 2 The First Department of Neurology, China-Japan Union Hospital of Jilin University, Changchun, China

**Keywords:** Phosphorylation, Mechanisms of disease

## Abstract

Mitochondrial fission is a critical physiological process in eukaryotic cells, participating in various vital activities such as mitosis, mitochondria quality control, and mitophagy. Recent studies have revealed a tight connection between mitochondrial fission and the mitochondrial metabolism, as well as apoptosis, which involves multiple cellular events and interactions between organelles. As a pivotal molecule in the process of mitochondrial fission, the function of DRP1 is regulated at multiple levels, including transcription, post-translational modifications. This review follows the guidelines for Human Gene Nomenclature and will focus on DRP1, discussing its activity regulation, its role in mitochondrial fission, and the relationship between mitochondrial fission and apoptosis.

## Facts

- The function of DRP1 in cells is precisely regulated at multiple levels including transcription and translation, etc.
- Under certain stress conditions, mitochondrial fission and apoptosis often occur concomitantly.
- DRP1 is the pivotal molecule at the crossroads between mitochondrial fission and apoptosis processes.

## Open questions

- In the process of apoptosis under varied conditions, which occurs earlier, mitochondrial fission or the change in the permeability of the mitochondrial outer membrane?
- What are the differences in the pathophysiological processes between apoptosis induced by mitochondrial fission and that induced by changes in mitochondrial permeability?
- Can the occurrence of apoptosis be regulated by adjusting mitochondrial fission and fusion (It may be a feasible therapy for the treatment of cancers and neurodegenerative diseases)?

## Introduction

As the “power plant” of eukaryotic cells [1], mitochondria not only provide energy support for various cellular activities but also participate in multiple biosynthetic and catabolic reactions, playing a crucial role in apoptosis and other cellular events [2, 3]. Mitochondria are organelles that constantly undergo dynamic changes by the means of fission and fusion at all times [4, 5]. And the dynamic balance between fission and fusion shapes the morphological structure of mitochondria, which in turn affects mitochondrial function, adapting to various demands of cells [6]. Mitochondrial fusion is mediated by proteins Mfn1 and Mfn2 involved in outer membrane fusion, and Opa1 involved in inner membrane fusion [7, 8]. In contrast, mitochondrial fission is mediated by molecules such as DRP1, Mff, Mid49/51, and Fis1 [9, 10], among which DRP1 stands in dominant position [11, 12].

Under stress or various pathological conditions, mitochondrial fission serves as the driving force inducing mitochondria to evolve towards “fragmented” state [13, 14], which excludes damaged ones from the mitochondrial system known as mitochondria quality control (MQC), and it also affects mitochondrial metabolism and mitophagy [15, 16]. Additionally, previous studies revealed that apoptosis often occurs concurrently with mitochondrial fragmentation [17], suggesting a connection between mitochondrial fission and apoptosis. And the connection may contribute to the processes of neurodegenerative diseases and tumors [18, 19]. Research on DRP1 will help to elucidate the mechanism of multiple diseases and provide new insights for their diagnosis and therapy.

## Characteristics of the DRP1 gene and molecular structure

**Fig. 1:**
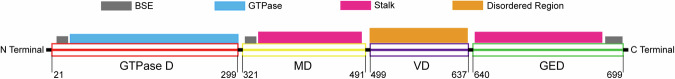
The structure of DRP1.

The gene encoding the DRP1 protein, *DNM1L*, is located at 12p11.21 and consists of 21 exons with a total length of 66,451 base pairs [20]. The encoded protein, Dynamin-related protein 1 (DRP1) has nine splice variants [21, 22]. And based on its functions, DRP1 can be divided into four domains structurally [12](Fig. 1): 1. GTPase domain: The domain is primarily responsible for binding and hydrolyzing GTP [23]; 2. Middle domain: It is mainly involved in the self-assembly and polymerization of DRP1 molecules [24, 25]; 3. Variable domain(VD): It participates in the regulation of DRP1 polymerization and influences the curvature of the DRP1 complex helix. Additionally, most of the post-translational modification sites of the DRP1 are located within the VD region [26, 27]; 4. GED domain: In the 3D-structure, this domain folds back and interacts with the GTPase domain to regulate and activate the GTPase activity of DRP1 [24, 28]. GTPase domain acts as the catalytic domain, while stalk is comprised of middle domain and GTPase effector domain (GED), which is consistent to other member of Dynamin superfamily proteins (DSPs) [12]. In addition, fission DSPs include an additional bundle signaling element (BSE) connecting the stalk and G domain [29]. What’s more, VD is the unique intervening sequence adjacent to the stalk [30].

## Mitochondrial fission

Mitochondrial fission is a complex process involving multiple factors. Although some mechanisms remain elusive, existed studies could outline the process to us. The mechanism involves the following steps: 1. Endoplasmic reticulum (ER) constrains mitochondria. At the site of mitochondrial fission, the ER-mitochondria contact interface, the ER-associated actin regulatory factor INF2 and Spire1C work together to facilitate actin polymerization [31, 32]. Myosin II participates by binding to actin filaments, constricting mitochondria into a tubular shape at the fission site [33]. Actin-binding proteins such as Cofilin, Cortactin, and the Arp2/3 complex may also be involved in this process [34]. 2. DRP1 receptor recruits DRP1. Current DRP1 receptors on the mitochondrial surface consist of Mff, Mid49/51, and Fis1. Although it is found in in vitro that Fis1 can bind to DRP1, knockout of Fis1 has little effect on the mitochondrial fission process. However, some studies have found that Fis1 participates in the formation of a complex containing DRP1, Mff and MAMs-related proteins, and then plays role in the stress-induced mitochondrial fission process [35]. Both Mff and Mids can recruit DRP1 independently, but given the absence of Mff having the most significant impact on mitochondrial fission, which suggests that Mff is thought to play a dominant role in DRP1 recruitment to mitochondria [36]. Studies on the functions of Mff and Mids revealed that they play a synergistic effect in mitochondrial fission. Cells lacking either Mff or Mids exhibit impaired mitochondrial fission ability, while cells lacking all three are incompetent to undergo mitochondrial fission [37]. Previous research has shown that Mff can recruit activated, oligomerized DRP1, while Mids inactive, dimerized DRP1 [38]. In a liposome model, it was found that the binding of Mff to DRP1 can upregulate DRP1’s GTPase activity, while the binding of Mid51 to the opposite [39]. This depicts a model that dimerized DRP1, upon binding to Mids, whose GTPase activity is restricted, ensures that DRP1 conjuncts with unhydrolyzed GTP consistently. Once DRP1 assembly is completed, Mff, which shares the same spatial localization with Mids [40], binds to DRP1 and activates its GTPase activity, facilitating the scission action at the fission site. Other studies have also reported that the assembly and recruitment of DRP1 at the mitochondrial fission site involve phosphorylation regulation at multiple sites of the DRP1 [41] and the recruitment and activation of polymerized actin, Myosin2, and INF2 by DRP1 [42].

## The regulation of DRP1 activity

The activity of the DRP1 is regulated at multiple levels and in complex manners, and the intricate regulatory network ensures the precise execution of DRP1 function temporally and spatially.

### Regulation at transcriptional and translational levels

The existing body of evidence regarding Drp1 transcriptional regulation is hitherto scarce. Some studies have reported that P53 bonds to the promoter region of DNM1L to upregulate DRP1 transcription, and repressing P53 impeded mitochondrial fission and the activation of apoptosis [43, 44]. Additionally, c-Myc also upregulated DRP1 expression at the transcriptional level through miR-373-3p, a process that played a role in the pathophysiology of hepatic cancer development [45].

At the translational level, the RNA-binding protein Hu antigen R(HuR) can bind to the 3’ untranslated region of DRP1 RNA, ensuring DRP1 translation, and knocking down HuR definitely downregulate DRP1 expression and promoting mitochondrial fusion [46]. The heterogeneous nuclear ribonucleoprotein A1 (hnRNP A1) also regulates DRP1 expression by interacting with DRP1 mRNA at its 3’UTR region directly, enhancing translation process without affecting mRNA stability [47]. Inhibition of hnRNP A1 results in mitochondrial fusion, while overexpression of hnRNP A1 promotes mitochondrial fission. Further research is needed to elucidate the mechanisms of DRP1 expression at the transcriptional and translational levels.

### Post-translational modifications

Recent studies have revealed that the regulation of DRP1 function is primarily achieved through post-translational modifications, among which phosphorylation is the most extensively studied one.

#### Phosphorylation modification

(1) The orderly temporal and spatial regulation of DRP1 function is essential for cell proliferation and development, and the phosphorylation modification of DRP1 plays crucial role in the mechanism.

It was found that during mitosis, the cyclin B1/Cdk1 complex phosphorylates DRP1 at the Ser-585 site to promote DRP1 aggregation on mitochondria membrane, maintaining a short rod-like shape of mitochondria, which facilitates their distribution to daughter cells. After mitosis, the mitochondria regain their reticular shape in daughter cells [48]. PINK1 phosphorylates DRP1 at the Ser616 site to contribute to the maturation of spinal dendrites and axons [49, 50], a critical process of neuron development. Under physiological conditions, CDK19/Cdk8 phosphorylates DRP1 at the Ser616 site to drive mitochondrial fission, maintaining the normal function of the mitochondrial system. What’s more, studies have found that CDK19/Cdk8 can rescue the DRP1 disfunction caused by PINK1 mutations, suggesting that mutations or deficiencies in CDK19/Cdk8 may be a cause of Parkinson’s disease [51]. These physiological roles are just the tip of the iceberg for DRP1 functions, and more details remain to be revealed.

(2) Under various stresses or pathological conditions, the phosphorylation status of DRP1 is a critical factor affecting mitochondrial function and is tightly related to apoptosis, which plays role in the onset and progression of multiple diseases.

Cyclin-dependent kinase (CDK) is an important enzyme in the cell cycle [52]. Current research has found that it plays a role in various diseases and pathophysiological processes by phosphorylating multiple sites on the DRP1. In the radiation-induced optic neuropathy, CDK5 phosphorylates DRP1 at the Ser616 site, promoting mitochondrial fission and affecting mitochondrial function, which is tightly associated with the progression of the disease [53–55]. Studies have also reported that in NMDA-induced neuron loss, CDK phosphorylates DRP1 at the Ser585 site, forcing mitochondrial fission [56], and interfering with this process can alleviate neuron death, reversing the pathophysiological process. In Alzheimer’s disease (AD) model, CDK5 also phosphorylate DRP1 at the Ser579 site, which promotes mitochondrial fission and enhances neuron sensitivity to Aβ, leading to neurodegenerative diseases [57, 58].

What’s more, varied kinases could phosphorylate specific sites on DRP1 (for instance the Ser616 site) participating in various pathophysiological processes. In the pathology of osteoarthritis (OA), the activation of TANK-binding kinase 1 (TBK1) and the following phosphorylation of DRP1 at the Ser616 site contribute to the progress of the disease [59]. In the pathological process of renal fibrosis, TGF-β-induced phosphorylation of DRP1 at the Ser616 site plays a significant role [60]. And existing research has found that in LPS-induced inflammatory responses, signal transducers and activators of transcription 2 (Stat2) phosphorylates DRP1 at the Ser616 site, promoting the accumulation of DRP1 on mitochondria, which plays an important role in macrophage differentiation [61]. Further, in various inflammatory responses involving macrophages, Protein Kinase C delta (δPKC)phosphorylates DRP1 at the Ser616 site, inducing mitochondrial fission [62]. It also found that mitogen-activated protein kinase 1 (MAPK1) phosphorylates DRP1 at the Ser616 site leaving fragmented mitochondria, which participates in the pathology of Huntington’s disease (HD) [63]. Under radiation stress, CaMKII phosphorylates DRP1 at the Ser-616 site, mediating the occurrence of mitochondrial fission, which plays a role in the apoptosis procedure of cells under radiation stress [64, 65].

Additionally, the phosphorylation of varied sites on DRP1 has been found in the progression of various diseases. IFN-β can phosphorylate and activate STAT5 that is an up-regulator of PGAM5 expression, and the following upregulation of PGAM5 promotes the dephosphorylation of DRP1 at the Ser643 site; and the dephosphorylation at the S643 site provides privilege for CaMKIIa to phosphorylate DRP1 at the Ser622 site, promoting mitochondrial fission [66], which participates in the pathological processes of multiple neurodegenerative diseases. Rho-associated, coiled-coil-containing protein kinases (ROCK1) phosphorylates DRP1 at the Ser600 site, promoting the mitochondrial fission [67], which promotes mtROS generation and apoptosis in glomerular cells, playing an important role in the pathology of microvascular lesions induced by hyperglycemia. In muscle cells, AMPK phosphorylates DRP1 at the Ser637 site, promoting mitochondrial fusion [68], which can counteract the mitochondrial fission and dysfunction under high-fat diet (HFD) treatment, and it takes part in the pathological process of fatty liver [69]. Glycogen synthase kinase 3β (GSK3β) can phosphorylate the Ser40/44 sites to promote mitochondrial fission, increasing neuron sensitivity to Aβ and promoting neuronal apoptosis [70]; however, under oxidative stress, it can phosphorylate the Ser693 site, promoting mitochondrial fusion and enhancing the stress resistant ability of cells to stress [71]. And c-Abl can phosphorylate threonine at the 266, 368, and 449 sites, driving mitochondrial fission and apoptosis [72], which contribute to the neuron loss under oxidative stress.

(3) In addition to kinases, phosphatases also play a significant role in the regulation of DRP1 activity.

ROCK1 upregulate the activity of PP1 and PP2A to dephosphorylate DRP1 at the Ser637 site, activating DRP1 activity and promoting its aggregation on mitochondria and resulting in mitochondrial fission [73–75]. In an MPTP-induced Parkinson’s disease (PD) model, ROCK1 also participates in the dephosphorylation of DRP1 at the Ser656 site, contributing to mitochondrial fission [76]. Phosphoglycerate mutase 5 (PGAM5) can dephosphorylate DRP1 at Ser-637 site, promoting the translocation of DRP1 to mitochondria, achieving mitochondrial fission [77], which plays an important role in the programmed necrosis. In RGCs cells, A-kinase anchoring protein 1 (AKAP1) conducts the dephosphorylation of DRP1 at the Ser637 site, promoting mitochondrial fission and participating in the pathophysiology of glaucoma [78].

In addition to the classic phosphorylation regulation of DRP1 by kinases or phosphatases, the state of DRP1 phosphorylation is also related to other types of modifications. Those modifications may alter the conformation of the DRP1, affecting the phosphorylation status of DRP1. For instance, PDI-induced S-nitrosylation of DRP1 can promote the phosphorylation of DRP1 at the Ser616 site, resulting in mitochondrial fission [79].

Under various conditions, multiple factors affect the phosphorylation status of amino acid residues at multiple sites of DRP1 through phosphorylation, dephosphorylation, or the altered conformation of DRP1, thereby regulating mitochondrial function and even affecting cell fate. These sites are distributed across different domains of the DRP1, and the Ser616 and Ser637 sites located in the GED region were extensively studied [26] (see the Table 1 below for details). However, the commonalities of these sites after phosphorylation have not been thoroughly elucidated.

**Table 1:** The modification sites and Kinases/Phosphatases.

Domain	Range	Sites	Kinase/Phosphatase
GTP	21–299	Ser40/44	GSK3β [70]
		Thr266	c-Abl [72]
MD	300–491	Thr368/449	c-Abl [72]
VD	499-637	Ser579	CDK5 [57, 58]
		Ser585	CDK [56]
		Ser600	ROCK1 [67]
		Ser616	PINK1 [49, 50];CDK19/Cdk8 [51]; CDK5 [53–55]; TBK1 [59]; TGF-β [60];STAT2 [61];δPKC [62, 127–129]; MAPK1 [63];CaMKII [64, 65];JNK [130]; ERK1/2 [63, 131–134];ROCK [67]
		Ser622	CaMKIIa [66]
		*Ser637*	AMPK [68];PKA [108, 135–138];CaMKIα [139];PKD [140];CaN [97, 141, 142]; *ROCK1* [74]*;PP1/PP2A* [73–75]*;PGAM5* [77]*; AKAP1* [78]*;*
GED	640–734	*Ser656*	*ROCK1* [76]
		*Ser693*	GSK3β [71]

*Sites*: underlined sites driving fission if phosphorylated; italicized sites driving fusion if phosphorylated.

*Kinases/Phosphatases*: underlined ones are kinases; italicized ones are phosphatases.

#### SUMOylation modification

Small ubiquitin-like modifier (SUMO) is a conserved molecule that conjugates to the lysine residue of substrates in the post-translational modification (PTM) [80]. There exist five SUMO isoforms in mammalian cells, named SUMO1, 2, 3, 4, and 5, with SUMO2 and SUMO3 sharing 96% homology. Current research suggests that sumoylation can promote the stability of DRP1 attaching to the mitochondrial surface and contribute to mitochondrial fission [81, 82]. Studies revealed that SUMO ligase MAPL SUMOylates DRP1 at K532, K535, K558, K568, K594, K597, K606, and K608 sites in VD region, promoting its anchoring on mitochondria and mitochondrial fission [83, 84]. In contrast, Sentrin/SUMO-specific protease 5 (SENP5) inhibits mitochondrial fission by de-sumoylating DRP1 [85]. However, SENP3 can promote DRP1 binding to MFF and induce mitochondrial fission, cytochrome c release, and apoptosis by desumoylating DRP1 [86–89]. Additionally, different SUMO modifications on the same substrate lead to different pathophysiological effects. Inhibiting SENP3 enhances the covalent binding of DRP1 to SUMO2/3, impeding the cytochrome c release and protecting against ischemia-induced cell death [89]. Overexpressing SUMO1 can promote mitochondrial fission and stabilize DRP1 on mitochondria [90], and SENP5 can reverse the mitochondrial fission induced by SUMO1 through deSUMOylating DRP1 [85].

Furthermore, ubiquitination can also affect the activity of DRP1. For example, Parkin promotes the degradation of DRP1 through ubiquitination [91], while the anaphase-promoting complex/cyclosome and its coactivator Cdh1 (APC/C) can regulate the mitochondrial fission during mitosis by ubiquitinating and degrading DRP1 [92]. Reversely, OTUD6A cleaves off ubiquitin residues and increases DRP1 stability in the cell [93].

## Relationship between mitochondrial fission and apoptosis

Mitochondrial fission is temporally and spatially correlated with apoptosis. Under physiological conditions, activation of about 3% DRP1 is sufficient to maintain the balance between fusion and fission of the mitochondrial system [94]. However, excessive activation of DRP1 and its aggregation on mitochondria can affect mitochondrial function and even initiate apoptosis [95, 96].

Existing studies indicate that inducing mitochondrial fission can promote apoptosis [97–99], while mitochondrial fusion may be a means for cancer cells to evade apoptosis induced by chemotherapy [100, 101]. Inhibiting DRP1 activity and mitochondrial fission, whether through chemical or genetic knockout methods, can exert anti-apoptotic effects [102–104]. In a study on lung adenocarcinoma, inducing mitochondrial fusion under chemotherapy can elevate chemo-resistant ability of cancer cells [105]; inhibiting DRP1 activity through inhibitors or genetic intervention can exert anti-apoptotic effects [103, 104](the most commonly used inhibitors of DRP1 listed in Table 2). In addition, in PINK1 double knockout and MPTP drug-induced PD models, inhibiting DRP1 activity significantly reduces the loss of substantia nigra neurons and promotes neuron survival [102].

**Table 2:** The most commonly used inhibitors of DRP1.

Inhibitors	Mechanisms
DRP1i27	Binding to the human isoform 3 of Drp1 and inhibiting the activity of GTPase [143].
P110	Inhibiting Drp1 enzyme activity and blocking Drp1-Fis1 interaction [144].
Mdivi-1	Attenuating the polymerization of DRP1 to higher order structures at early stages of fission by inhibiting GTPase activity [103, 145].
Anti-inflammatory agent 49	Inhibiting the GTPase activity of Drp1 and Drp1-Fis1 interaction [146].
Drpitor1a	Suppressing the GTPase activity of Drp1 without inhibiting the GTPase of dynamin 1 [147].
MB0223	Selectively inhibiting the oligomeric form of DRP1 [148].
Honokiol DCA	Blocking the phosphorylation of DRP1 at Ser-616 [149].

It is currently believed that cells with fragmented mitochondria are more sensitive to apoptosis-related stimuli [106, 107], which may be attributed to the inefficient energy synthesis state of fission mitochondria [106, 108]. Inhibiting mitochondrial fission and promoting mitochondrial fusion can enhance the efficiency of the mitochondrial electron transport chain and increase oxidative phosphorylation capacity [109–111], increasing the stress-resistant ability, which is consistent with our previous study [112]. In addition, the mitochondrial fission can induce mitochondrial MOMP and genomic instability, which may also be vital factor involved [113].

**Fig. 2:**
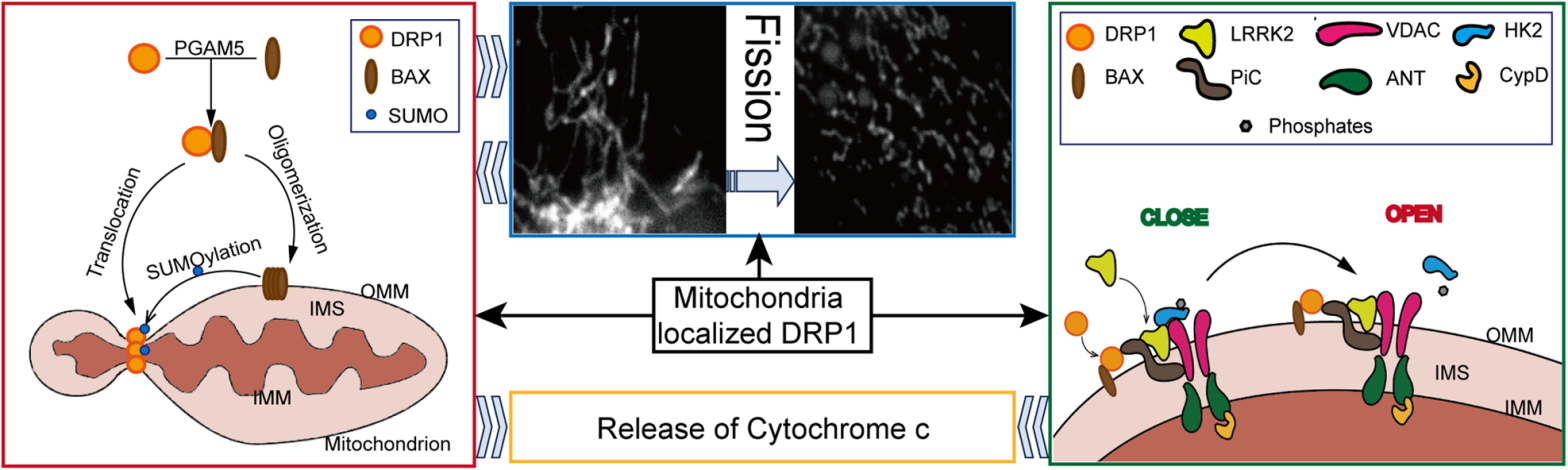
The function of mitochondria localized DRP1. Mitochondrial-localized DRP1 not only functions in mitochondrial fission but also influences the apoptotic process by interacting with BCL2 family members and regulating the mitochondrial permeability transition pore (mPTP).

Further, research has found that DRP1 can promote the release of mitochondrial cytochrome c and the activation of Caspases [104]; DRP1 interacts with Bcl2 family, promoting the translocation of pro-apoptosis molecule BAX to mitochondria [114] or directly suppressing the activity of anti-apoptosis Bcl2 [115] to execute apoptosis. Some studies suggest that DRP1 can bind to the N-terminal domain of BAX, which simultaneously promotes the activity of BAX and DRP1, on the one hand, promoting the oligomerization of BAX to drive the apoptosis, and on the other hand, accelerating the translocation of DRP1 to mitochondria to promote mitochondrial fission [116], and this interaction between BAX and DRP1 may be achieved in a PGAM5 dependent manner [117]. In addition, during the apoptosis, BAX and BAK can stabilize DRP1 on the mitochondrial outer membrane by promoting the sumoylation of DRP1 [118], and the colonization of DRP1 on the mitochondrial membrane in turn conducts mitochondrial fission and the apoptosis [119, 120], thus forming a positive feedback process to induce apoptosis, which may be one of the reasons why mitochondrial fission and apoptosis are associated(Fig. 2). In other studies, with DRP1 knockout, a single BAX oligomer ring cannot launch the release of cytochrome c from inter mitochondrial membrane space (IMS) [121, 122]. Suppressing DRP1 activity can counteract mitochondrial fragmentation, impede the release of cytochrome c, and prevent apoptosis, and this process is independent of the translocation of BAX to mitochondria [123], suggesting that DRP1 not only participates in the activation of BAX but also in the MOMP. It should be noted that during the apoptosis, DRP1 can anchor to the mitochondrial outer membrane in a BAX/BAK-dependent manner, and which does not trigger mitochondrial fission [84], suggesting that although the apoptotic role of DRP1 is closely related to mitochondrial fission, there still exists independence between each other to some extent.

In addition, Duan et al. found that DRP1 conduct the opening of the mitochondrial permeability transition pore (mPTP) to induce apoptosis. Under hypoxic conditions, first, DRP1 recognizes BAX and PiC and contacts with mPTP; then, DRP1 recruits and inactivates LRRK2, leading to the inactivation and dissociation of HK2, which induces an alteration in the conformation of mPTP and result in excessive opening [124]. Moreover, other studies suggest that the formation of the complex of PGAM5-L, BAX, and DRP1 is a fundamental step in the occurrence of intrinsic apoptosis [117]. To the contrary, some studies found that mitochondrial fission exhibits anti-apoptotic properties [125, 126]. For example, in brain tumor research, knocking down DRP1 in tumor-initiating cells can suppress cell growth and induce apoptosis [125], which may be attributed to specific cell types or physiological conditions (Fig. 2).

It is currently believed that during the mitochondrial fission, activated DRP1 can promote BAX activity, alter mitochondrial membrane permeability, and induce pro-apoptotic substances release, promoting apoptosis.

## Conclusion

As the key player in the process of mitochondrial fission, the activity of DRP1 is regulated at multiple levels, in various ways to manipulate mitochondrial function precisely and adjust it to the demands of cells under varied physiological conditions, external stress, and pathological processes. Firstly, DRP1 is an essential molecule for normal physiological processes such as mitosis and mitochondrial quality control. Secondly, the mitochondrial fission is closely related to the apoptosis; and under external/inner stress such as ischemia-hypoxia or chemotherapy, DRP1 act as the balancer, which determines the fate of cells and plays vital role in the progress of diseases. In summary, elucidating the regulatory mechanisms of DRP1 under different conditions is of great significance, as it will help clarify the coordinated interaction between various cellular events and provide new perspectives for the diagnosis and treatment of multiple diseases. However, to achieve this goal, more in-depth research is still needed.
